# Vancomycin-induced acute generalized exanthematous pustulosis (AGEP) masquerading septic shock—an unusual presentation of a rare disease

**DOI:** 10.1186/s40560-015-0114-3

**Published:** 2015-11-10

**Authors:** Sagger Mawri, Tarun Jain, Jainil Shah, Gina Hurst, Jennifer Swiderek

**Affiliations:** 1 Department of Internal Medicine, Henry Ford Hospital, 2799 W Grand Blvd, Detroit, MI 48202 USA; 2 Department of Internal Medicine and Emergency Medicine, Henry Ford Hospital, 2799 W Grand Blvd, Detroit, MI 48202 USA; 3 Department of Pulmonary and Critical Care, Henry Ford Hospital, 2799 W Grand Blvd, Detroit, MI 48202 USA

**Keywords:** Acute generalized exanthematous pustulosis, Vancomycin, Steroids, Septic shock, Drug eruption

## Abstract

Acute generalized exanthematous pustulosis (AGEP) is a rare cutaneous adverse reaction characterized by acute sterile pustular eruptions, mostly induced by medications. Antibiotics are the most commonly implicated drugs; however, there have only been two previous reports of vancomycin-induced AGEP in the literature. In this case, we present the clinical course of a 56-year-old man who was admitted to the intensive care unit with an unusually severe form of AGEP mimicking septic shock, which developed after the recent use of vancomycin. Despite cessation of the offending agent, our patient continued to clinically decline with development of worsening skin eruptions and hemodynamic instability necessitating vasopressor support. The patient promptly responded to systemic steroid therapy with complete resolution of AGEP. In addition to highlighting the implication of vancomycin in AGEP, we herein discuss the clinical presentation, diagnosis, and management of AGEP, particularly in severe cases admitted to the intensive care unit.

## Background

Acute generalized exanthematous pustulosis (AGEP) is a rare disorder characterized by an acute-onset severe cutaneous reaction manifesting as several non-follicular pustules on a diffuse erythematous edema [1, 2]. It is usually a drug-related reaction, with antibiotics most commonly implicated [2]. It is typically a self-limited condition that resolves spontaneously with cessation of the offending agent. While it typically has systemic features such as fever and leukocytosis, it rarely causes multi-organ involvement [3]. Herein, we describe a unique case of vancomycin-induced AGEP in a middle-aged male presenting with distributive shock and multi-org.

## Case presentation

A 56-year-old man with newly diagnosed glioblastoma multiforme underwent craniotomy and tumor resection followed by external beam radiation and concurrent chemotherapy with temozolomide. His post-operative course was complicated by an epidural abscess, which was initially drained. Fluid cultures revealed methicillin-resistant streptococcus epidermitis (MRSE). The patient subsequently underwent resection of the abscess followed by cranioplasty with placement of a vancomycin-impregnated titanium mesh to repair the skull defect. He was discharged home on a course of steroid therapy for vasogenic edema noted on post-operative brain MRI as well as an 8-week course of intravenous vancomycin administered via a peripherally inserted central catheter. His steroid taper was dexamethasone 4 mg po twice a day for 3 days, then 2 mg twice a day for 3 days, then 2 mg once a day for 3 days, and 1 mg once a day for 3 days.

The patient tolerated the antibiotic infusion well. Two weeks later, having completed his dexamethasone taper 2 days prior, he presented to the emergency department complaining of a diffuse red, itchy rash. He also reported experiencing fevers reaching 38 °C and chills over the past day. He denied any face or tongue swelling, nausea, vomiting, abdominal pain, or diarrhea. He reported no prior skin rashes or allergic reactions and no new medications or other antibiotics. He was not on any other medications except the IV antibiotic infusion.

**Fig. 1:**
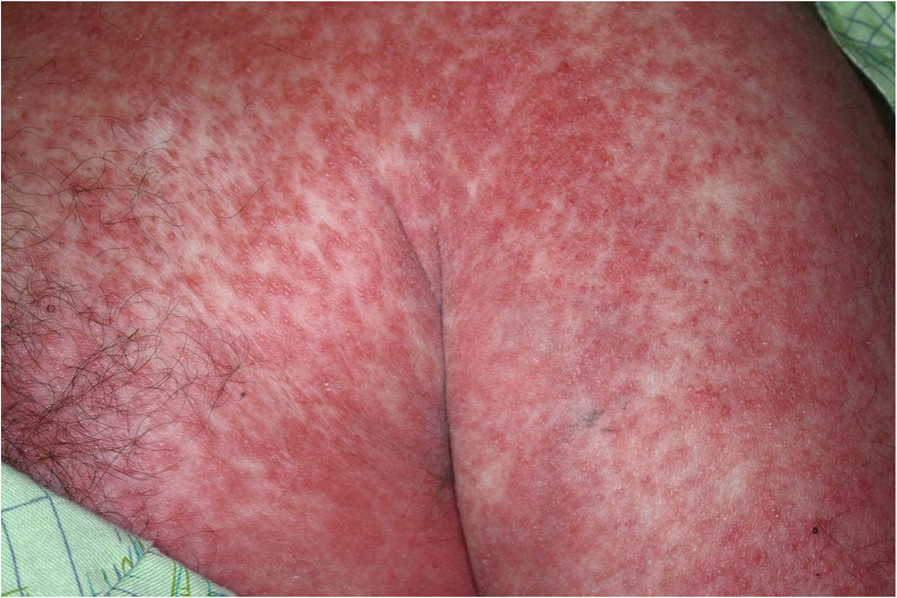
Day 3 of hospital admission, before initiation of systemic steroid therapy. Numerous small studded pustules on an erythematous background with numerous erythematous macules and papules coalescing into plaques are noted in various body parts

On presentation, the patient was febrile at 38.4 °C, tachycardic at 130/min, tachypneic at 32/min, hypotensive at 88/55 mmHg, and saturating 96 % on room air. Physical examination revealed numerous small studded pustules on an erythematous background on the forehead, frontal scalp, cheeks, trunk, upper and lower extremities, and axilla, with numerous erythematous macules and papules coalescing into plaques noted on the back, trunk, and extremities (Fig. 1). There was no evidence of mucous membrane involvement, lymphadenopathy, or organomegaly. There was no neck stiffness, changes in speech, numbness, or weakness. Initial laboratory studies revealed a normal white blood cell count (9200/μL) with a left shift (22 % bands) and without eosinophilia, an elevated lactate level (3.2 mmol/L), normal TSH, and minimally elevated C-reactive protein and erythrocyte sedimentation rate. His serum creatinine was 1.2 mg/dL with GFR of 89 mL/min. His liver function tests were mildly elevated (AST 56 IU/L; ALT 50 IU/L).

The patient was aggressively resuscitated with multiple intravenous crystalloid fluid boluses. Vancomycin was stopped and intravenous linezolid was started. Chest radiograph was normal. Brain MRI did not show evidence of leptomeningeal enhancement or signs of recurrent infection. Abdominal ultrasound was unremarkable. The patient was admitted to the infectious disease ward to undergo further work-up. Dermatology was consulted and skin biopsies were obtained. He was started on topical steroids for the skin rash, which was suspected to be acute generalized exanthematous pustulosis. However, the patient continued to have fevers, and he remained tachycardic, tachypneic, and hypotensive despite multiple intravenous fluid boluses. His urine output became diminished and his mental status became depressed. His antibiotic coverage was broadened to include intravenous cefepime and metronidazole in addition to linezolid. He was transferred overnight to the medical intensive care unit for escalation of care and suspected septic shock.

**Fig. 2:**
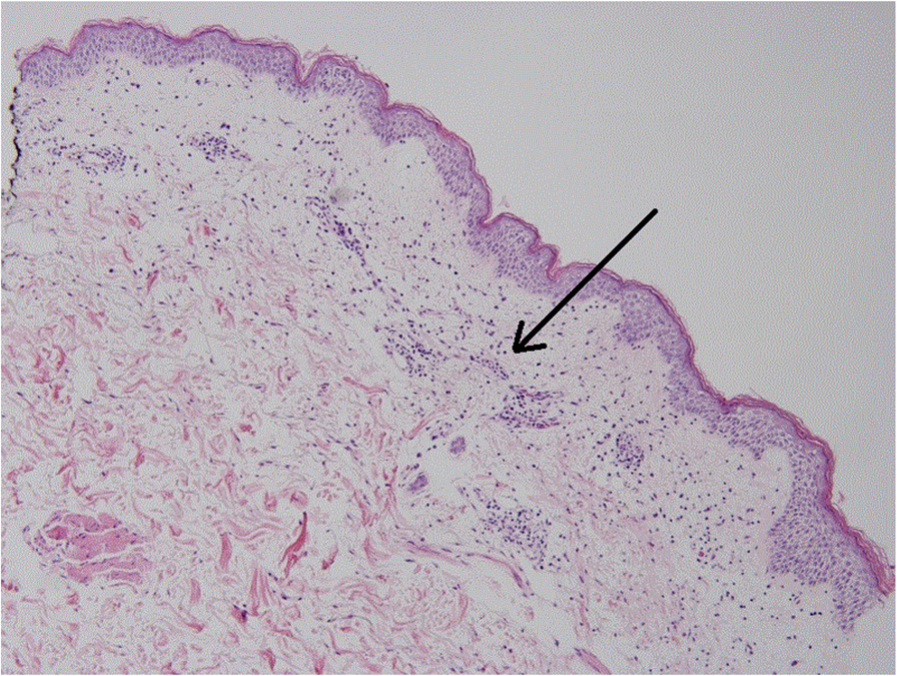
Histopathology of skin biopsy revealing spongiotic epidermis with focal parakeratosis, exocytosis, and spongiotic vesicles, along with papillary dermal edema, superficial dermal perivascular inflammatory infiltrate, and mixed dermal interstitial inflammation with eosinophils (H&E, ×10). The arrow points to the "papillary dermal edema and superficial dermal perivascular inflammatory infiltrate"

While in the intensive care unit, the patient was continued on empiric broad-spectrum antibiotic coverage and given additional intravenous fluid boluses. He continued to have tachycardia, tachypnea, increased work of breathing, and fevers reaching 39 °C. The skin examination worsened, with more diffuse skin involvement and development of edema of the face. Laboratory investigation revealed bandemia (28 %). He developed increasing somnolence, reduced urine output, signs of volume overload with elevated central venous pressure (14 mmHg), and increasing oxygen requirements. Vasopressors were started for hypotension. The infectious work-up was negative to date, including multiple sets of negative blood cultures. Histopathological examination of the skin punch biopsy revealed sub-corneal and intraepidermal pustules, widespread spongiosis, and neutrophil infiltration around the vessels in the papillary dermis—findings consistent with acute generalized exanthematous pustulosis (Fig. 2). In light of the patient’s worsening clinical status and apparent lack of response to topical steroids, he was started on high-dose steroids with Solu-Medrol 80 mg IV daily per recommendations from the dermatology consult team. The patient’s weight was 79.5 kg; thus, steroid dosing was determined using 1 mg/kg dosing calculation, which has been shown to be an effective starting dose for severe AGEP and similar dermatologic conditions.

**Fig. 3:**
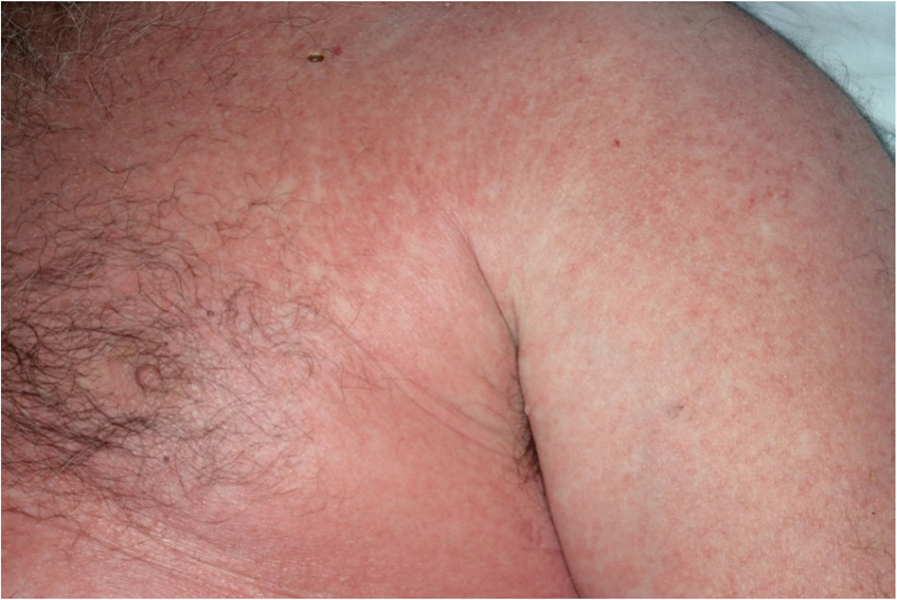
Day 4 of hospital admission, after initiation of systemic steroid therapy. Significant regression of skin eruptions noted

The patient responded remarkably well to the systemic steroids. He had rapid clinical improvement and within hours became afebrile, was weaned off of the vasopressors, and had complete resolution of his tachycardia and tachypnea. His mental status improved, urine output increased, and respiratory status normalized. Within 24 h, the skin rash had regressed significantly (Fig. 3). The antibiotics were discontinued, except for linezolid. Intravenous Solu-Medrol was transitioned to prednisone 80 mg oral daily. The patient was discharged home in stable condition on a prednisone taper, planned to be titrated down by 10 mg weekly. He was also discharged on linezolid 600 mg oral twice a day to complete his initial antibiotic course. Six months later, the patient has completed his antibiotic and steroid treatment courses and continues to do well. The patch test for vancomycin was deemed unnecessary by the dermatology consult team because the patient was not on any home medications aside from steroids and vancomycin; thus, the only obvious culprit was vancomycin. In vitro testing such as macrophage migration inhibition factor (MIF) test or mast cell degranulation (MCD) test were not performed, as these are unavailable at our institution.

### Discussion

AGEP is a rare disorder characterized by the acute development of sterile, non-follicular pinhead-sized pustular eruptions overlying an edematous erythema [1, 2]. Characteristically, the skin reaction arises rapidly within a few hours and tends to resolve quickly within several days after cessation of the offending agent or may resolve spontaneously. The rash mostly begins in the intertriginous areas or in the face then spreads diffusely and is often described as a burning or itching sensation. In about 20 % of cases, mucous membranes are involved but tend to be mild and usually limited to the oral cavity [1].

The incidence of AGEP has been approximated at 1 to 5 cases per million annually [4]. More than 90 % AGEP cases are related to drugs, with viruses comprising the next most common trigger. The most commonly implicated drugs are antibiotics, including pristinamycin, ampicillin, amoxicillin, macrolides, and quinolones. Other drugs include calcium channel blockers and antifungal agents [2]. Although sulfa drugs can cause many skin reactions, they are usually not implicated in AGEP [5].

AGEP commonly presents with high fevers and neutrophilia concurrently occurring with the skin eruptions, thus often misinterpreted as an acute infectious disease. It tends to occur suddenly within 1 to 2 days of exposure to the offending agent and is generally self-limited when the causative agent is eliminated. The skin pustules typically begin on the face and within hours spread to the trunk and limbs and tend to coalesce. Multi-organ involvement is rare and highly atypical of AGEP [3, 6]. Histological evaluation of the involved skin shows spongiform subcorneal and intraepidermal pustules, papillary dermal edema, and neutrophilic infiltration around papillary dermal blood vessels [1].

Important differential diagnoses for AGEP include Stevens-Johnson syndrome (SJS) and toxic epidermal necrolysis (TEN). The systemic features of AGEP usually manifest early, whereas SJS/TEN tends to have a slower manifestation, usually after 3 weeks, and characteristically presents with blisters and severe mucus membrane involvement. Furthermore, the drugs implicated in SJS/TEN are usually different, such as antiepileptic agents or allopurinol [7].

A useful algorithm for the diagnosis of AGEP was proposed by Sidoroff et al. [1]. The validation score combines clinical and histological features, such as the type and distribution of the skin rash, presence of leukocytosis, histological findings, and the timing of the skin reaction. A score of 8 to 12 is definitive for the diagnosis of AGEP. Applying the AGEP validation score resulted in a score of 11 in our patient, confirming the diagnosis.

Typically self-limiting in nature, AGEP tends to resolve spontaneously with cessation of the offending agent and no further measures are usually required. The use of steroids is not routinely required and if indicated, usually topical steroids suffice in providing clinical improvement [6]. Systemic steroids have been used on rare occasions when AGEP presents atypically with severe systemic involvement [8–10].

Our case has several unique features. First, the onset of AGEP was delayed for a couple of weeks after initiation of the offending antibiotic, and this was most likely because the patient was on concurrent steroid therapy for his vasogenic cerebral edema. Immediately following completion of the steroid therapy, and hence loss of its anti-inflammatory effects, AGEP was abruptly unmasked.

Second, in contrast to most reported cases of AGEP, our case demonstrates an atypical presentation with severe systemic involvement and multi-organ failure mimicking septic shock. The clinical presentation continued to deteriorate despite cessation of the offending antibiotic. Because the patient was only on a brief (12 days) and tapered-down course of steroid therapy, it was not felt that his hemodynamic instability was secondary to adrenal insufficiency, and thus, this was not worked up with laboratory testing.

Third, the presence of antibiotic-impregnated intracranial mesh added another layer of complexity to our case, as it was not evident what role this played in our patient’s clinical picture. While there is scarce data in current literature on this particular matter, it has been reported that systemic absorption and side effects in antibiotic-impregnated orthopedic implants are usually minimal [11]. However, given the continued clinical decline of our patient after cessation of the systemic antibiotic, one may speculate about the potential contribution of antibiotic release from the intracranial mesh.

Fourth, the role of systemic steroids in the management of AGEP is rarely indicated and topical steroids usually suffice if the disorder does not resolve with cessation of the offending agent [3]. However, as our case illustrates, despite the use of topical steroids, the patient’s skin eruptions progressed rapidly coinciding with rapid clinical decline, hemodynamic instability, and multi-organ involvement necessitating vasopressors. Administration of high-dose intravenous steroids resulted in rapid clinical improvement.

Finally, vancomycin as a cause of AGEP is exceptionally rare. To the best of our knowledge, there have only been three previously reported cases of vancomycin-induced AGEP in the literature [8, 9, 12]. One case included another confounding antibiotic that is more commonly implicated in AGEP, and thus, the causation relationship was equivocal. The second case presented typically with resolution of AGEP following cessation of vancomycin, with no additional required interventions such as steroids. And the third case presented atypically with a severe AGEP course similar to our case and required systemic steroids. In the latter case, the patient required only several days of systemic steroids with complete clinical recovery. However, because of the presence of vancomycin-impregnated intracranial mesh in our patient with unclear clinical significance, a prolonged systemic steroid taper was chosen for our patient.

## Conclusion

AGEP is a rare disorder characterized by acute onset of characteristic skin eruptions, often triggered by certain medications. This condition is typically self-limiting in nature and tends to resolve spontaneously with cessation of the offending agent. The use of steroids is not routinely required. However, as this case illustrates, AGEP could present atypically with aggressive multi-organ involvement and hemodynamic compromise mimicking septic shock despite cessation of the offending agent. In such cases, early systemic steroid administration appears to provide immediate hemodynamic stabilization and clinical improvement.

## Consent

Written and verbal consent was obtained from the patient for publication of this case report and use of accompanying images. A copy of the written consent is available for review by the Editor in Chief of this journal.

## Abbreviations

AGEP: acute generalized exanthematous pustulosis
MRSE: methicillin-resistant streptococcus epidermitis
SJS: Stevens-Johnson syndrome
TEN: toxic epidermal necrolysis
